# Artificial intelligence applications in psychoradiology

**DOI:** 10.1093/psyrad/kkab009

**Published:** 2021-07-02

**Authors:** Fei Li, Huaiqiang Sun, Bharat B Biswal, John A Sweeney, Qiyong Gong

**Affiliations:** Huaxi MR Research Center (HMRRC), Department of Radiology, West China Hospital of Sichuan University, Chengdu 610041, Sichuan, P.R. China; Research Unit of Psychoradiology, Chinese Academy of Medical Sciences, Chengdu 610041, Sichuan, P.R. China; Functional and Molecular Imaging Key Laboratory of Sichuan Provience, Department of Radiology, West China Hospital of Sichuan University, Chengdu 610041, Sichuan, P.R. China; Huaxi MR Research Center (HMRRC), Department of Radiology, West China Hospital of Sichuan University, Chengdu 610041, Sichuan, P.R. China; Research Unit of Psychoradiology, Chinese Academy of Medical Sciences, Chengdu 610041, Sichuan, P.R. China; Functional and Molecular Imaging Key Laboratory of Sichuan Provience, Department of Radiology, West China Hospital of Sichuan University, Chengdu 610041, Sichuan, P.R. China; Department of Biomedical Engineering, New Jersey Institute of Technology, Newark, NJ 07102, USA; The Clinical Hospital of Chengdu Brain Science Institute, MOE Key Lab for Neuroinformation, University of Electronic Science and Technology of China, Chengdu 610054, Sichuan, P.R. China; Huaxi MR Research Center (HMRRC), Department of Radiology, West China Hospital of Sichuan University, Chengdu 610041, Sichuan, P.R. China; Department of Psychiatry and Behavioral Neuroscience, University of Cincinnati, Cincinnati, OH 45219, USA; Huaxi MR Research Center (HMRRC), Department of Radiology, West China Hospital of Sichuan University, Chengdu 610041, Sichuan, P.R. China; Research Unit of Psychoradiology, Chinese Academy of Medical Sciences, Chengdu 610041, Sichuan, P.R. China; Functional and Molecular Imaging Key Laboratory of Sichuan Provience, Department of Radiology, West China Hospital of Sichuan University, Chengdu 610041, Sichuan, P.R. China

**Keywords:** Psychoradiology, magnetice resonance imaging, brain, artificial intelligence, machine learning, deep learning, graph neural network

## Abstract

One important challenge in psychiatric research is to translate findings from brain imaging research studies that identified brain alterations in patient groups into an accurate diagnosis at an early stage of illness, prediction of prognosis before treatment, and guidance for selection of effective treatments that target patient-relevant pathophysiological features. This is the primary aim of the field of Psychoradiology. Using databases collected from large samples at multiple centers, sophisticated artificial intelligence (AI) algorithms may be used to develop clinically useful image analysis pipelines that can help physicians diagnose, predict, and make treatment decisions. In this review, we selectively summarize psychoradiological research using magnetic resonance imaging of the brain to explore the neural mechanism of psychiatric disorders, and outline progress and the path forward for the combination of psychoradiology and AI for complementing clinical examinations in patients with psychiatric disorders, as well as limitations in the application of AI that should be considered in future translational research.

## Overview

Psychiatric disorders are heterogeneous in etiology, symptoms, and treatment response. They are primarily diagnosed based on specialized doctors’ experience and subjective judgments in mental health services. Accurate differential diagnosis of psychiatric disorders, especially before illness onset and early in the course of illness, is particularly important for early effective treatment and early prediction of prognosis. The sole reliance on subjective clinical observation and patient reports limits precision in these efforts. Therefore, there is a pressing need to identify objective biomarkers to assist clinicians in making accurate clinical diagnoses, identifying subgroups of individuals with similar behavioral syndrome features that may require differential therapeutics, and predicting disease prognosis (Ivleva *et al*., 2020). This approach has significantly advanced clinical practice in many fields of medicine, but its application in clinical psychiatry is only beginning. Magnetic resonance imaging (MRI) -based biomarkers, providing direct data about the anatomy and function of the target organ (brain), form perhaps the most promising option for developing clinically actionable biomarkers (Li *et al*., 2020b).

Psychoradiology is an emerging field that applies medical imaging technologies in the evaluation of patients presenting with psychiatric disorders (Gong, 2020). So far, much work in this area has involved research investigation of brain features that differ between patients and healthy individuals to develop models of illness pathophysiology, rather than establishing the basis for clinical application (other than to rule out neurological abnormalities that may contribute to behavioral problems). As advances continue in showing the use of MRI for clinical diagnosis and clinically useful subtyping of heterogeneous psychiatric syndromes, the process of translation of research findings to clinical practice is beginning to evolve.

Psychoradiology relies on quantitative analysis of imaging data rather than traditional visual inspection of images, which is important because the brain alterations associated with psychiatric illness are subtle. MRI, together with other medical imaging techniques such as positron emission tomography and electroencephalography, is the mainstay of psychoradiology. As a result, advances in this field require interdisciplinary collaborations among radiologists, psychiatrists, psychologists, and computer scientists (Huang *et al*., 2019; Lui *et al*., 2016; Rosenberg *et al*., 1997; Yin *et al*., 2020). Rather than focusing on behavioral features currently used for psychiatric diagnosis, imaging methods focus on the translational investigation of underlying neuropathological mechanisms to provide objective and biological diagnostic and prognostic biomarkers of major mental disorders to advance clinical practice. With the application of artificial intelligence (AI), psychoradiology may provide useful information for the diagnosis, prediction of prognosis, prevention, and treatment of psychiatric disorders.

Because multimodal imaging provides compact data of still uncertain clinical relevance for psychiatry, AI implementation is a promising strategy for identifying the optimal use of different imaging metrics for different clinical purposes. The surge in AI development is made possible by the evolution of quantitative approaches for using large amounts of data and powerful computer systems that can rapidly process big data. There have been many areas in which AI has been successfully used in medicine to support decision-based medical tasks through knowledge/data-intensive computer-based solutions that improve a human care provider's performance. Adoption of AI in medical imaging can result in faster diagnoses and reduced errors, and, as a result, the interest in the development of AI research and its clinical implementation has skyrocketed.

The use of innovative biomarkers, considered clinically together with assessment of clinical symptoms, cognition, and laboratory tests, is a promising pathway forward to improve differential diagnoses and individualized patient care for psychiatric disorders. The application of AI methods has incorporated standard evaluation metrics and advanced biomarkers to develop and evaluate computer-based decision aids. The use of AI approaches in the evolving field of psychoradiology might transform empirically supported advances in the development of AI-based tools into routine workflow and administration of psychoradiological examinations and clinical practice. The diagnostic methods and tactics of diagnosis and prediction with medical imaging might be improved by the use of AI. Different imaging modalities in conjunction with AI might provide key information for multiple disorders and different clinical questions. In this context, the next necessary step is to evaluate AI methods and decision aids to establish and then validate their use in the clinical settings, where improved patient care and outcomes are the ultimate indicators of success.

## Psychoradiological Findings

### Differences in brain MRI measurements

Multimodal MRI can identify biomarkers for psychiatric disorders that reveal the subtle neural mechanisms of these disorders relevant to clinical decision-making. Taking adult antipsychotic-naive first-episode patients with schizophrenia as an example (Table 1), gray matter structural alterations have been most robust within thalamo-cortical networks, whereas altered brain function has been most pronounced in fronto-parietal and default-mode networks. These findings indicate that regional anatomical and functional brain alterations revealed by MRI are significantly dissociated during the early course of schizophrenia before the initiation of first treatment with antipsychotic treatments (Gong *et al*., 2016). The short-term (6 weeks) and long-term (1 year) effects of atypical antipsychotics on brain function have an intricate pattern of changes both toward normalization and that shift metrics further from normal function. This may reflect a pattern of drug effects that includes both a reduction in illness-related pathologies as well as changes that may be compensatory or represent adverse effects of the drug therapies (Anticevic *et al*., 2015; Keedy *et al*., 2014; Li *et al*., 2016; Lui *et al*., 2010).

**Table 1:** Summary of reviewed psychoradiological studies with adult samples.

Study	Group comparison	Sample size	Public dataset	Image modality	Imaging features	Results
(Lui *et al*., 2010)	SZ (longitudinal) vs HC	SZ: 34 (before and after 6-week treatment) HC: 34	No	rfMRI	ALFF, seed-based FC	Increased ALFF in the prefrontal, parietal, and superior temporal cortex and caudate nucleus were found in SZ after 6-week atypical antipsychotic treatment and correlated with clinical improvement in positive symptoms. After treatment, widespread FCs of these regions (seeds) were decreased in SZ, which were correlated with the increased ALFF values in all seed areas, but not with symptomatic ratings
(Meng *et al*., 2019)	SZ (longitudinal) vs HC (longitudinal)	SZ: 35 (before and after 6-week treatment) HC: 19 (before and after 6-week follow-up)	No	DTI	FA, RD, AD	Reduced FA (primarily due to decreased AD) occurred after acute 6-week treatment along with clinical recovery in patients, which suggested possible adverse effects contributing to widespread reduced white matter microstructural integrity, including bilateral corona radiata, posterior thalamic radiation, left posterior limb of the internal capsule, and body of the corpus callosum
(Anticevic *et al*., 2015)	SZ (longitudinal) vs HC	SZ: 129 (before treatment), 25 (1-year follow-up) HC: 106	No	rfMRI	GBC	During early illness course and before drug treatment, increased GBC in the SZ group relative to HC was found around the MPFC, which was positively correlated with the severe overall symptom severity in patients and could classify patients and controls with 63% accuracy. After 1 year of treatment, the increased PFC GBC reduced and normalized to the level of HC and there were positive relationships between the magnitude of PFC GBC normalization and improvement in clinical symptoms
(Li *et al*., 2016)	SZ (longitudinal) vs HC (longitudinal)	SZ: 20 (before and after 1-year treatment) HC: 16 (before and after 1-year follow-up)	No	rfMRI	ALFF, seed-based FC	The decreased ALFF in right IPL and OFC, increased ALFF in right occipital gyrus, and FC between bilateral IPL in patients with SZ at baseline were normalized to the level of HC after 1-year atypical antipsychotic treatment. However, the lower ALFF in the bilateral thalami and ventral MPFC, the higher ALFF in the bilateral precuneus and the right amygdala, and increased FC between OFC and dorsal MPFC in SZ than control at baseline remained significantly at 1-year follow-up
(Zhang *et al*., 2015)	SZ vs HC	SZ: 25 (never-medicated patients with long-term SZ) HC: 33	No	sMRI	cortical thickness, gray matter volume	The never-treated patients exhibited reduced cortical thickness in bilateral ventral MPFC, left superior temporal gyrus, and right pars triangularis than HC. In these regions, patients showed a greater rate of age-related decline of cortical thickness than HC. Greater cortical thickness in the left superior parietal lobe was also observed in these patients with a reduced rate of cortical thinning relative to control participants. The volumetric analysis showed grey matter enlargement of the putamen bilaterally, and volume reduction of the right middle temporal gyrus and right lingual gyrus in patients, but age-related changes in these regions did not differ from HC
(Yao *et al*., 2019)	never-treated SZ vs antipsychotic-treated SZ vs HC	SZ: 21 (never-treated long-term illness)SZ: 26 (treated long-term illness) HC: 24	No	rfMRI	Graph theoretical analysis based on FC	Never-treated patients had decreased global efficiency and shortest path length of the brain functional networks than HC and treated patients, which were correlated with the total symptom severity in never-treated SZ. There were no differences in network organization characteristics between HC and treated patients. Lower nodal efficiency of left pre-/postcentral gyri was shown in both patient groups and never-treated patients demonstrated additional lower nodal efficiency of bilateral putamen/caudate and right amygdala/hippocampus than treated patients and HC
(Zhang *et al*., 2019)	SZ vs HC	SZ: 39HC: 31	No	DTI	FA	Compared to HC, patients with SZ showed reduced FA in the body of corpus callosum, superior longitudinal fasciculus, posterior thalamic radiation, and corona radiata. In the SZ group, reduced FA in the left posterior thalamic radiation and corpus callosum was negatively correlated with greater fasting plasma glucose level, and the interaction between glucose and FA in the left corpus callosum, longitudinal fasciculus, and corona radiata was associated with the deficit cognitive performance assessed by MATRICS Consensus Cognitive Battery
(Wu *et al*., 2019)	SZ vs HC	SZ: 44HC: 44	No	sMRI	Cortical thickness, surface area, and cortical and subcortical gray matter volume	In patients with SZ, significantly critical associations were found between the increased serum cytokines level of IL-6 and IL-10 and the cortical thickness mainly located in the inferior frontal and superior temporal cortices that were decreased than controls
(Lizano *et al*., 2020)	SZ vs BD vs HC	SZ spectrum: 79Psychotic BD I: 61HC: 60	No	sMRI	cortical thickness and subcortical volume	Psychosis probands had higher levels of CRP, TNFα, VEGF, and IL6 than HCs, and BD had lower CRP and IL6 levels than SZ. In probands, the CRP was mainly negatively associated with putamen volume and cortical thickness in the left postcentral, supramarginal, and transverse temporal cortices, and right cuneus and precuneus; and TNFα was positively correlated with the right medial OFC and middle temporal thickness and the left thalamus volume. In an exploratory analysis, a subgroup (biotype) with higher inflammatory levels had increased subcortical volume and thickness mainly in frontal and temporal cortices than the proband subgroup with low inflammation
(Yu *et al*., 2019b)	MDD vs HC	MDD: 189HC: 39	No	rfMRI	Network FC	Compared to controls, MDD patients showed decreased within-network connectivity in the frontoparietal network (FPN), dorsal attention network (DAN), and cingulo-opercular network (CON); increased within-network connectivity in the default mode network, salience network, sensorimotor network, and visual network. By using canonical correlation analysis, significant associations were found in MDD between a history of childhood trauma and a multivariate pattern of seven within- and between-network connectivities mainly including FPN, DAN, and CON, etc.

Abbreviations: BD, bipolar disorder; MDD, major depression disorder; SZ, schizophrenia; HC, healthy control; sMRI, structural MRI; ts-fMRI, task state functional MRI; rfMRI, resting-state functional MRI; ALFF, amplitude of low-frequency fluctuations; FC, functional connectivity; GBC, global brain connectivity; FA, fractional anisotropy; RD, radial diffusivity; AD, axial diffusivity; MPFC, medial prefrontal cortex; IPL, inferior parietal lobule; OFC, orbitofrontal cortex; IL, interleukin; CRP, C-reactive protein; TNFα, tumor necrosis factor-alfa; VEGF, vascular endothelial growth factor.

The therapeutic implications of regional and network-level brain function evolving in schizophrenia require further study but may already be relevant for clinical application. To explore brain changes over the course of illness independent of acute and long-term effects of antipsychotic treatment, cross-sectional studies in chronically ill schizophrenia patients that were never treated with antipsychotics over 5 to 20 years of illness duration have found an accelerated age-related decline in cortical thickness of prefrontal and temporal cortex (Zhang *et al*., 2015), suggesting a neuro-progressive process in schizophrenia. Compared with those patients with similar illness duration who had long-term treatment with antipsychotic medication, the never-treated compared to treated patients showed more severe functional brain alterations (Yao *et al*., 2019). Those differences suggest that although there may be early adverse effects of antipsychotic medications on brain anatomy and function (Lui *et al*., 2010; Meng *et al*., 2019), the long-term effects of antipsychotic treatment may protect against age-related disease effects on brain global and nodal health (involving the amygdala, hippocampus, and striatum) (Yao *et al*., 2019). The extent of these beneficial and adverse treatment effects varies across patients in ways that may be dose- or drug-class related, so their use in clinics could potentially guide optimized therapeutics.

In childhood (Table 2), atypical brain developmental trajectories are associated with a range of adverse behavioral and cognitive effects (Pavuluri and Sweeney, 2008), as is the case in autism spectrum disorder (ASD) (D'Cruz *et al*., 2016; Takarae *et al*., 2008), pediatric mood disorders (Schenkel *et al*., 2012), and attention deficit hyperactivity disorder (ADHD) (Li *et al*., 2014c; Passarotti *et al*., 2010). Structural MRI data obtained from more than 30 centers worldwide showed that children with ADHD had smaller intracranial volume (Boedhoe *et al*., 2020), lower cortical surface area (mainly in frontal, cingulate, and temporal regions), and thinner fusiform gyri and temporal pole cortical thickness (Hoogman *et al*., 2019). In normal maturation, local brain connectivity decreases while long-range connectivity increases (Posner *et al*., 2020). In this context, the increased functional connectivity (FC) between the prefrontal cortex and striatum and decreased long-range connectivity in fronto-parietal and fronto-cerebellar networks in pediatric ADHD revealed by a previous study (Li *et al*., 2014a) might reflect delayed or dysfunctional maturation. Findings from psychoradiological research showing abnormal brain development complement the genetic, cognitive, and environmental information in comprehensively characterizing neurodevelopmental alterations and associated disabilities of ADHD (Posner *et al*., 2020).

**Table 2:** Summary of reviewed psychoradiological studies with pediatric and adolescent samples.

Study	Group comparison	Sample size	Public dataset	Image modality	Imaging features	Results
(D'Cruz *et al*., 2016)	ASD vs HC	ASD: 17HC: 23	No	ts-fMRI	Functional activation	Although there were no behavioral performance differences in reversal learning tasks, the ASD group exhibited reduced activation in brain regions related to cognitive decision-making processes (i.e. frontal cortex) and reinforcement learning processes (i.e. ventral striatum) than controls when choice outcome after reversal was uncertain
(Passarotti *et al*., 2010)	BD vs ADHD vs HC	BD: 15ADHD: 17HC: 14	No	ts-fMRI	Functional activation	The two pediatric patient groups both showed slower response time in an emotional valence Stroop task and greater activation in the dorsolateral prefrontal cortex and parietal cortex than HC for negative versus neutral words. The BD group showed greater activation in the ventrolateral prefrontal cortex than both groups of ADHD and HC
(Li *et al*., 2014c)	ADHD vs HC	ADHD: 33HC: 27	No	ts-fMRI	Functional activation	During the working memory task, patients with ADHD showed significantly higher activation in the bilateral globus pallidus and the right hippocampus than controls and a positive correlation between the activation of the left globus pallidus and the reaction time to correct responses of the task
(Boedhoe *et al*., 2020)	ADHD vs ASD vs OCD vs HC	ADHD: 2271ASD: 1777OCD: 2323HC: 5827	ENIGMA	sMRI	Subcortical volumes, cortical thickness, surface area	No shared structural differences were found among all three disorders than controls. Children with ADHD showed smaller hippocampal volumes than matched OCD, and children and adolescents with ADHD had smaller intracranial volume than age-matched three other groups. Adult ASD had increased frontal cortical thickness than age-matched three other groups
(Hoogman *et al*., 2019)	ADHD vs HC	ENIGMA-ADHD (ADHD: 2246, HC: 1934) Generation-R (general population: 2707)	ENIGMA-ADHD, Generation-R	sMRI	Cortical thickness, surface area	Lower surface area in frontal, cingulate, and temporal regions and lower cortical thickness in the fusiform gyrus and temporal pole were found in children with ADHD than controls. The surface area of the caudal middle frontal gyrus and middle temporal gyrus was negatively associated with attention problems in the general population
(Li *et al*., 2014a)	ADHD vs HC	ADHD: 33HC: 32	No	rfMRI	ALFF, seed-based FC	Relative to healthy controls, patients with ADHD showed increased ALFF in bilateral globus pallidus and right dorsal superior frontal gyrus and decreased ALFF in the left orbitofrontal cortex and ventral superior frontal gyrus, along with decreased long-range FC in the frontoparietal and frontocerebellar networks and increased FC in the frontostriatal circuit that correlated with the executive dysfunction

Abbreviations: BD, bipolar disorder; HC, healthy control; ENIGMA, Enhancing Neuro Imaging Genetics through Meta-Analysis; sMRI, structural MRI; ts-fMRI, task state functional MRI; rfMRI, resting-state functional MRI; ALFF, amplitude of low-frequency fluctuations; FC, functional connectivity.

### Clinical relevance

In clinical practice, early course patients who will go on to have persistent debilitating symptoms and poor response to therapy are difficult to identify before choosing a course of treatment. Identification of pretreatment MRI features that predict such outcomes might guide an earlier consideration of alternative/adjunctive pharmacotherapy, behavioral treatments, or interventional treatment for modulating neural circuits with deep brain stimulation (DBS). In this latter therapy, identified specific brain regions in which abnormalities are associated with psychiatric disorders could serve as treatment targets. This framework has obvious appeal for translational drug development programs. DBS has been approved by the Food and Drug Administration in the USA for movement disorders and humanitarian use in severe treatment-nonresponsive patients with obsessive-compulsive disorder (OCD). The striatum, subthalamic nucleus, and internal capsule have been selected as potential DBS targets for OCD, but the response rate and side effects vary among different patient groups (Kohl and Baldermann, 2018). The Food and Drug Administration has approved focused transcranial magnetic stimulation for depression. With technical advances and established clinical benefit from new therapies such as transcranial magnetic stimulation and MR-guided focused ultrasound, noninvasive and imaging-guided therapeutic procedures offer promise now as secondary treatment options. However, their safety and broader efficacy require further investigation (Xu *et al*., 2019).

Although psychoradiology-based biomarkers have been used to guide interventional therapy in neurology, their use remains limited in psychiatry and the underlying pathophysiology of psychiatric disorders is not well understood. Multiple factors have been implicated, including genetic or epigenetic factors, alterations in immune-inflammatory response systems including the immune-regulatory reflex system, glycometabolism, and environmental factors (Li *et al*., 2020c). A voxel-wise and genome-wide association study showed that a missense mutation in gene *SLC39A8* was associated with larger gray matter volume in the putamen, and such an association was significantly weakened in schizophrenia (Luo *et al*., 2019). Interaction between glucose metabolism abnormality and white matter dysconnectivity in the corpus callosum and longitudinal fasciculus may lead to cognitive impairment in first-episode drug-naive schizophrenia (Zhang *et al*., 2019). Similarly, altered cytokine levels, especially IL-6, have been associated with the abnormal cortical thickness of bilateral Broca's area and superior and middle temporal gyrus, providing neuroimaging evidence to support the relationship between peripheral cytokines and the cerebral cortex in schizophrenia (Lizano *et al*., 2020; Wu *et al*., 2019). Children with prenatal alcohol exposure, compared with those without, exhibit greater psychopathology, attention deficits, and impulsiveness, and larger brain regional volume and surface area (Lees *et al*., 2020). For adult patients with MDD, a history of traumatic childhood experiences and current depressive symptoms has been associated with different within- and between-network FCs involving the dorsal attention network, frontoparietal network, and subcortical regions (Yu *et al*., 2019b). Further neurobiological and neurobehavioral studies are needed to link psychoradiological findings with genes, cytokine, and environment-induced changes at the molecular level, along with parallel efforts to establish the clinical utility of psychoradiological examinations in the practice of psychiatry.

### Limitations

Despite the rise of psychoradiological evidence demonstrating illness-related brain abnormalities in psychiatric disorders during the past decades, the impact of imaging research on the clinical practice of psychiatry remains very limited. The latest version of the Diagnostic and Statistical Manual of Mental Disorders does not include neuroimaging indicators as factors relevant for diagnostic evaluation. The main reason is that in the early phase of psychoradiology research, most studies adopted case-control designs comparing groups of patients with a specific disorder with healthy community controls. While this facilitated biological understanding of the illness, it did not advance clinical diagnostics as the clinical evaluation and diagnosis remained the gold standard against which imaging markers were compared. From the perspective of developing the clinical field of psychoradiology, this method of research design has limitations. First and foremost, by definition, imaging data cannot “do better than” clinical psychiatric evaluations in diagnostic accuracy when the clinical diagnosis is the gold standard. It can only do worse or in the optimal case match the clinical diagnosis, but even in that ideal case, it would not increase the diagnostic accuracy achieved by standard clinical methods. Second, there is well recognized and extensive biological heterogeneity in common psychiatric syndromes, and this is not resolved by statistical methods focusing on patient–control group comparison. Using between-group brain image differences for mechanistic understanding and clinical diagnosis requires a rigorous exploration of the coefficient of variation of the brain image characteristics, the normality or bimodality of feature distribution, and patterns of association of MR features in both patients and controls. The imaging characteristics used for clinical diagnosis need to have large differences between groups and small intragroup variations to have sufficiently low false positive or false negative rates to be clinically useful. And last, case-control study designs do not address clinically relevant questions of psychiatry, name approaches that can add to what clinical evaluations provide to enhance patient care.

## Applications of AI in Psychoradiology

### Supervised and unsupervised learning approaches

Machine learning is a subset of AI applications that learns and adjusts parameters by itself to perform a specific task with increasingly greater accuracy. Machine learning methods can be divided into supervised learning and unsupervised learning. MR features that have been evaluated with machine learning algorithms include gray matter structure properties with cortex volume, thickness and area surface, white matter diffusion properties, and FC. Supervised learning algorithms can summarize rules or patterns from existing labeled data and form a discriminant model to predict or classify new data (Li *et al*., 2014b). Notably, using the support vector machine (SVM) method and functional MRI data acquired from more than 1000 patients with schizophrenia, functional striatal abnormalities (including spontaneous functional activity, intra- and extra-striatal FC) can distinguish individuals with schizophrenia from controls with an accuracy of 80% (sensitivity of 79.3% and specificity of 81.5%) (Li *et al*., 2020a). The combination of three modalities (functional MRI, diffusion tensor imaging, and structural MRI data) and SVM have identified the vital role of basal ganglia-thalamus-cortex circuitry in distinguishing schizophrenia patients from healthy controls with high accuracy of 91.75% (Zhao *et al*., 2020). In addition to ongoing efforts to increase the accuracy of MRI data to correctly classify patients and controls, current psychoradiological research is focusing more on the prediction of long-term clinical outcomes and the prediction of patients’ response to various therapeutics. Cao *et al*. used SVM and demonstrated that functional connections between the superior temporal cortex and other cortical regions can achieve individual-level diagnosis with 78.6% accuracy and treatment prediction with 82.5% accuracy in schizophrenia (Cao *et al*., 2020). FC within the default mode network and the visual network could predict the post-treatment Yale-Brown Obsessive-Compulsive Scale score identified by least absolute shrinkage and a selection operator regression model, and FC predicted treatment response with 70% accuracy, in patients with OCD (Reggente *et al*., 2018).

Unsupervised learning is used to explore possible patterns in datasets based on the distribution of characteristics in the data and is usually used to identify discrete heterogeneity, or subgroups, in large patient samples. For example, one active line of research has aimed to identify potential patient subgroups in the schizophrenia population by using specific imaging manifestations to differentiate each subtype to address the issue of neurobiological heterogeneity and refine diagnostic nosology. Using a single imaging modality data and agglomerative hierarchical clustering analysis, Sun *et al*. showed two distinct patterns of white matter abnormalities that exist at the early phase of schizophrenia, one having global abnormalities and more severe negative symptoms (Sun *et al*., 2015). Subsequently, one of the biggest multicenter studies in the USA, the Bipolar-Schizophrenia Network for Intermediate Phenotypes consortium identified three patient biotypes using multivariate taxometric analyses (*k*-means clustering) based on brain function biomarkers (neuropsychological, oculomotor, and event-related potential data) (Clementz *et al*., 2016), and this was further validated using brain structural imaging biomarkers and clinical features (Ivleva *et al*., 2017; Kelly *et al*., 2021). Recently, another study conducted spectral clustering and found two subgroups of first-episode drug-naive schizophrenia patients, where one subgroup exhibited functional hypoconnectivity among brain regions in salience network, default-mode network, and central executive network and had more severe clinical symptoms, while another subgroup with hyperconnectivity and greater deficits in cognitive flexibility (Liang *et al*., 2021). These studies all found different subtypes of patients in schizophrenia using biomarker approaches, and suggest differential etiologies that might be best treated by different therapies. While they represent a promising strategy for resolving neurobiological heterogeneity in the schizophrenia syndrome, replications with other patient samples are needed to confirm the findings, and studies are needed to establish the clinical utility of the MRI-based subgroup delineations.

### Limitations

Although some psychoradiological studies implementing machine learning have achieved satisfactory classification/prediction accuracy, limitations should be noted. First of all, regarding the data quality, the magnetic nonuniformity can lead to image distortion and signal loss at the border between brain areas and air when scanning the echo-planar imaging sequence for diffusion and functional MR images (Jezzard and Balaban, 1995). Funded by the National Institutes of Health of the USA, the Human Connection Project collects and shares human brain data, and recommends an advanced MR scanning protocol (Van Essen *et al*., 2012). Their protocol is designed with a series of complementary imaging parameters and auxiliary correction sequences, using a pair of phase-encoding reversed spin-echo scans with identical geometry to generate a field map for distortion correction of data derived from the use of an echo-planar imaging sequence (Glasser *et al*., 2013). As an emerging technique in deep learning introduced in 2014, generative adversarial networks (GAN) (Goodfellow *et al*., 2014) could partially address this data quality issue of brain images by generating synthesized images, such as between MR sequences (Yu *et al*., 2019a), with improved quality (Hagiwara *et al*., 2019) and segmentation (Hamghalam *et al*., 2020). A newly published study used GAN to generate 3T* images from original 1.5T T1-weighted brain images and found that the quality of the generated (3T*) images was better than the original ones, including higher signal to noise ratio. Then the authors trained fully convolutional networks (FCN) for classification and generated a 3T* images-based FCN classifier that yielded a higher accuracy for discriminating patients with Alzheimer's disease (AD) from healthy controls than the performance of the FCN model based on the 1.5T images (Zhou *et al*., 2021). However, analogous studies that have implemented the generated images from GAN for psychoradiological analyses are limited.

Second, model overfitting in machine learning often occurs due to characteristics including small sample size and high dimensions of brain imaging features, raising important concerns about replicability and feature optimization. Further, some studies consider feature selection as a preprocessing step with all available data (training and testing data, even as well as validating data) before model training, followed by cross-validation as a verification step after model training. The consequence of this procedure is that in subsequent cross-validation, although the test data is not directly involved in the training, it has influenced the training process during the feature selection, potentially making the estimated performance falsely higher than the actual performance of the model. For such selection bias, the impact is crucial for the accuracy of psychoradiological studies typically performed with relatively modest sample size and high feature dimensions. Feature selection nested in the cross-validation loop iteratively is recommended to deal with this issue.

## Deep Learning

Deep learning is a family of machine learning methods that adapt themselves to extract patterns from data to solve complex problems with higher accuracy, and have broken benchmark records in speech recognition, image recognition, and natural language processing. This is currently one of the most popular fields in machine learning. Deep learning can automatically discover the optimal feature representation from raw data, avoiding the subjectivity of feature extraction and selection in conventional machine learning, especially in the field of psychoradiology where the brain areas with abnormalities are unknown and a priori knowledge of the neural mechanisms of psychiatric disorders is uncertain (Vieira *et al*., 2017). Deep learning refers to the strategy of using multilayer neural networks to handle difficult tasks, whose inspiration comes from models of how the human brain processes information (Kriegeskorte, 2015). The ability to achieve higher orders of complexity and abstraction makes deep learning more suitable for exploring human brain image data that have a complex relationship between structure and function than traditional shallow models of machine learning (Vieira *et al*., 2017).

For example, as summarized in Table 3, using a deep learning algorithm, the brain surface area features of 6 to 12-month-old infants at high familial risk of autism could significantly predict the diagnosis of autism at 24 months, with a positive predictive value of 81% and a sensitivity of 88%. Investigators in that study demonstrated that the surface area of the superior frontal gyrus, postcentral gyrus, and inferior parietal gyri contributed significantly to the identification of ASD cases, indicating that brain structure changes occur even before the period when autistic behaviors are first emerging (Hazlett *et al*., 2017). This of course offers promise for early detection of illness to permit early behavioral interventions that can more effectively improve clinical outcomes. Using the ADHD-200 dataset consisting of 973 individuals, Chen and colleagues developed a multichannel deep neural network model to analyze combined features, i.e. a fusion of the multiscale brain functional connectome and personal characteristic data. They achieved an accuracy of 78.3% for ADHD detection, with a specificity of 84.2%, a sensitivity of 70.0%, and an area under the receiver operating characteristic curve (AUC) of 0.82 (Chen *et al*., 2019). Among all features, the middle frontal gyrus, inferior orbitofrontal cortex, and fusiform gyrus significantly contributed to the classification of patients and controls. Based on the multimodal data including cortical and subcortical structures from volumetric MRI scans and clinical information of 417 participants obtained from the Alzheimer's Disease Neuroimaging Initiative dataset, an interpretable deep learning strategy linked a FCN to a multilayer perceptron to generate high-resolution disease probability maps for neurologist-level diagnosis accuracy of AD. They achieved a mean AUC value of 0.996, which was validated on three independent cohorts successfully (Qiu *et al*., 2020). Chang and colleagues (Chang *et al*., 2020) presented a novel ensemble clustering method using deep learning to identify subtypes across the psychotic-affective disorder spectrum in a trans-diagnostic sample of major psychiatric disorders (MPDs), including 217 with major depressive disorder, 193 with schizophrenia, and 171 with bipolar disorder. Two subtypes were identified using the whole-brain amplitude of low-frequency (ALFF) data from functional MRI within the sample of MPDs, in which archetypal MPDs (60%) had increased ALFF in frontal regions and decreased ALFF in posterior brain areas, and atypical MPDs (40%) were characterized by decreased frontal and increased posterior ALFF. Their findings suggest that functional imbalance between frontal and posterior is a core trans-diagnosis biomarker differentiating subtypes of MPDs that could have implications for diagnosis and therapeutics.

**Table 3:** Summary of reviewed deep learning studies.

Study	Group comparison	Sample size	Public dataset	Image modality	Input features	Methods	Performance
(Hazlett *et al*., 2017)	HR (neg) vs HR-ASD	HR (neg): 91HR-ASD: 15	NDAR	sMRI	Surface area and cortical thickness	DL (Autoencoder) + SVM	Accuracy = 0.94, Positive predictive value = 0.81, Sensitivity = 0.88, Specificity = 0.95
(Chen *et al*., 2019)	ADHD vs HC	ADHD: 246HC: 346	ADHD200	rfMRI	Functional connectome and personal characteristic data	mcDNN	Accuracy = 0.783, AUC = 0.82, Sensitivity = 0.700, Specificity = 0.842
(Qiu *et al*., 2020)	AD vs HC	ANDI (AD: 188, HC: 299) AIBL (AD: 62, HC: 320) FHS (AD: 29, HC: 73) NACC (AD:209, HC: 356)	ANDIAIBLFHSNACC	sMRI	T1-weighted full MRI volumes and clinical data	FCN + MLP	ANDI (Accuracy = 0.968, AUC = 0.996, Sensitivity = 0.957, Specificity = 0.977) AIBL (Accuracy = 0.932, AUC = 0.974, Sensitivity = 0.877, Specificity = 0.943) FHS (Accuracy = 0.792, AUC = 0.876, Sensitivity = 0.742, Specificity = 0.808) NACC (Accuracy = 0.852, AUC = 0.954, Sensitivity = 0.924, Specificity = 0.810)
(Chang *et al*., 2020)	Identify MPD subtypes	SZ: 193; BD: 171;MDD: 217; HC: 363	No	rfMRIsMRIDTI	ALFF, cortical thickness, FA	DL + clustering	Identified two major ALFF-based subtypes: archetypal MPDs (60%) and atypical MPDs (40%). The two subtypes differed in genetic, multimodal MRI, and clinical characteristics
(Zhou *et al*., 2021)	AD vs HC	ADNI: 1.5T (HC: 229, AD: 188) ADNI: 3T (HC: 47, AD: 35) NACC: 1.5T (HC: 356, AD: 209) AIBL: 1.5T (HC: 93, AD: 14)	ADNINACCAIBL	sMRI	sMRI scans	GAN + FCN	ADNI: 1.5T (Accuracy = 0.840, Sensitivity = 0.736, Specificity = 0.921) AIBL: 1.5T (Accuracy = 0.887, Sensitivity = 0.631, Specificity = 0.926) NACC: 1.5T (Accuracy = 0.816, Sensitivity = 0.674, Specificity = 0.899) ADNI: 3T* (Accuracy = 0.821, Sensitivity = 0.741, Specificity = 0.890) AIBL: 3T* (Accuracy = 0.929, Sensitivity = 0.714, Specificity = 0.962) NACC: 3T* (Accuracy = 0.843, Sensitivity = 0.739, Specificity = 0.904)
(Yang *et al*., 2019)	BP vs HC	BP: 59HC: 47	No	rfMRIsMRI	Functional connectome and anatomical features	GAT	Accuracy = 0.820, F1-score = 0.828, Precision = 0.795, Recall = 0.867
(Ma *et al*., 2019)	ADHD vs HC Preclinical AD vs Prodromal AD	ADHD 200 (ADHD: 269, HC: 487) ANDI (Preclinical AD: 276, Prodromal AD: 171)	ADHD200 ANDI	ADHD200 (rfMRI) DNI (DTI)	ADHD200 (functional brain connectivity) ANDI (structural connectivity)	MGNN	ADHD200 (Accuracy = 0.649, Precision = 0.566, Recall = 0.649) ANDI (Accuracy = 0.774, Precision = 0.772, Recall = 0.774)

Note: HR (neg) means infants at high family risk for ASD but did not meet diagnostic criteria for ASD at 24 months of age; HR-ASD means infants at high family risk for ASD and met clinical criteria for ASD at 24 months of age; 3T* represents the GAN model generated 3T* images from original 1.5T images; Precision = true positive/(true positive + false positive); Recall = true positive/(true positive + false negative); F1-score = (2 × Precision × Recall)/(Precision + Recall).

Abbreviations: HR, high risk; NDAR, National Institutes of Health (NIH) National Database for Autism Research; sMRI, structural MRI, DL, deep learning;HC, healthy control; rfMRI, resting-state functional MRI; mcDNN, multichannel deep neural network; ANDI, Alzheimer's Disease Neuroimaging Initiative; AIBL, Australian Imaging, Biomarker and Lifestyle Flagship Study of Ageing; FHS, Framingham Heart Study; NACC, National Alzheimer's Coordinating Center; MLP, multilayer perceptron; MPD, major psychiatric disorder; BD, bipolar disorder; MDD, major depressive disorder; DTI, diffusion tensor imaging; ALFF, amplitude of low-frequency fluctuations; FA, fractional anisotropy; GAT, graph attention network; MGNN, multi-resolution graph neural network.

These studies provide promising proof of concept demonstration that deep learning is a very useful and powerful tool. However, applying these frameworks might be difficult for clinical researchers without deep learning expertise. There are millions of parameters in the deep neural networks that need to be determined during training, such as the number of nodes of each layer, the activation function of each node, how many layers should be used, etc. Due to the complexity of these tasks, the automated deep learning framework that emerged in the area of computer science can help make deep learning ready to use for clinicians (Faes *et al*., 2019). At present, the application of automated deep learning methods in brain imaging data of psychiatric disorders is still facing many difficulties and challenges. In addition to the large number of parameters that need to be learned in training, the human brain image itself contains very high dimensions of features. Moreover, the human brain has large variability between individuals as well as subtle differences between patients with psychiatric disorders and healthy controls. Therefore, a large amount of training data and powerful computing resources are required. With the fast development of graphical processing units, deep learning on neuroimaging will become less time consuming in the future. At present, how many samples are essential for deep learning in neuroimaging studies remains uncertain. If a deep neural network is trained on data with high dimensions and limited sample size, the risk of a model with overfitting is high. However, with the establishment of large standard brain imaging databases in psychiatric disorders through multi-center collaborations, and the development of computer hardware, deep learning is likely to be one of the most promising directions for psychoradiology.

## Graph Neural Network

**Figure 1:**
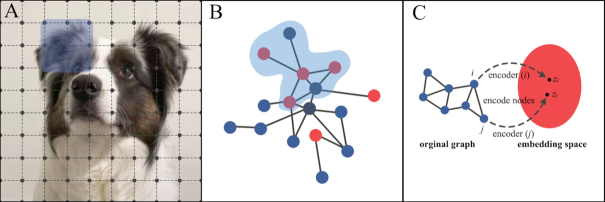
(A) 2D image in Euclidean space. The 2D convolution takes the weighted average of pixel values of the central node along with its neighbors. The neighbors of a node are ordered and have a fixed size. (B) Graph in non-Euclidean space. Different from the 2D image, the neighbors of a node in the graph are unordered and variable in size. (C) Illustration of graph embedding. The goal of graph embedding is to learn an encoder, which maps nodes to a low-dimensional embedding space.

**Figure 2:**
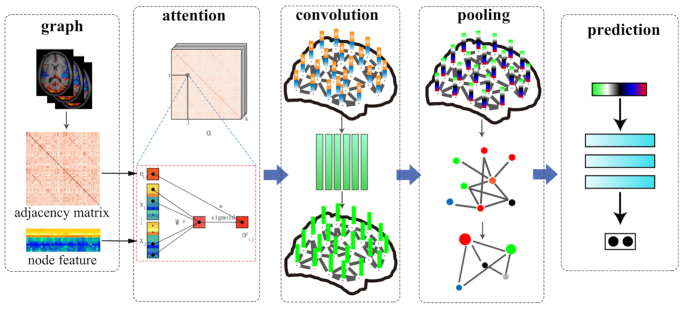
An example of a GNN model. (step 1: graph) Input of graphs. The graph is represented by an adjacency matrix and the set of node features. (step 2: attention) Graph attention layer. The graph attention layers take edge and node features as input. Using the attention mechanism, we can obtain the attention coefficients that indicate the importance of node *j*’s features to node *i*. This propagation step is optional. Here are other alternative propagation types such as convolution and gate mechanism. (step 3: convolution) Graph convolution layer. This layer extracts each node's hidden representation by aggregating features of its neighbors. (step 4: pooling) Pooling layer. Coarsen graphs into subgraphs that represent higher graph-level representations. (step 5: prediction) Multilayer perceptron for final prediction of the graph classification.

In recent years, deep learning has been successfully employed in many domains. Although deep learning can effectively extract an optimal representation of Euclidean data, there are also a large amount of data existing in the form of graphs. Examples of an image in Euclidean space and a graph in non-Euclidean space are shown in Fig. 1. Graphs are a data structure comprised of nodes (typically brain regions in neuroimaging studies) and edges (e.g. brain structural and functional connections). In a graph, each node is naturally defined by its own features and the relationship with other nodes. Previous graphic studies on neuroimaging converted brain structural or functional connectomes into binary or weighted network matrices by sparsity thresholds (Li *et al*., 2018). Then a series of topological parameters were calculated and treated as the subsequent input features in post analysis. This kind of feature construction method, which relies on manual intervention, may inevitably lead to information bias and loss of the graph. To address these issues, the concept of graph neural network (GNN) was first proposed by Scarselli and colleagues (Scarselli *et al*., 2009), which is a deep learning-based method that extends existing neural networks for processing data represented in graph domains. The first motivation of GNN has its roots in convolutional neural networks (CNN), which can extract multi-scale localized spatial features and compose them to construct latent representations (LeCun *et al*., 2015). However, CNN can only work on Euclidean data such as images and texts. Another motivation of GNN comes from the concept of graph embedding (Cui *et al*., 2019). As shown in Fig. 1C, graph embedding aims to represent graph nodes in low-dimensional vectors, preserving both information about the relative positions of the nodes in the graph and nodes self-content information. Given these backgrounds, GNN is proposed, which can model input or output consisting of features and their relationships collectively and train a classifier to predict the label of a graph (Fig. 2).

Regarding the application of GNN to psychoradiology, Yang and colleagues (Yang *et al*., 2019) developed an interpretable edge-weighted graph attention network (EGAT) framework combining anatomical features and functional connectivity measures to classify patients with bipolar disorder and healthy controls. They achieved a classification accuracy of 82%, which outperformed other models including random forest and SVM. By using an attention mechanism, this model revealed multiple interactive patterns among default mode, fronto-parietal, and cingulo-opercular networks underlying bipolar disorder. Ma and colleagues (Ma *et al*., 2019) proposed a novel GNN with a multi-resolution representation of the graph for identifying disease-specific variations in brain functional connectivity networks of ADHD and structural connectivity networks of patients with AD. Their multi-resolution framework surpassed other conventional graph methods in classification and identifying disease-specific brain connectivity patterns associated with ADHD or AD.

Although GNN has had considerable success in many fields, we should realize that GNN models have some limitations (Wu *et al*., 2020). Traditional GNN is not efficient enough to update the hidden states of nodes iteratively for a fixed point, and GNN uses the same parameters for different layers, which is not a hierarchical feature extraction process. Besides, compared with deep learning that can stack hundreds of layers to obtain better performance, GNNs are usually shallow with several layers. More importantly, the informative features of the edges cannot be represented effectively in the original GNN (Jie *et al*., 2018). Future research directions of GNNs include designing real deep GNNs to tackle more complex tasks, developing specific models to handle heterogeneity and diversity of graphs, and applying optimal representation methods that balance graph integrity and algorithm efficiency.

Due to its superior performance, deep learning on graphs is a promising and fast-developing research field in psychoradiology, and GNN has been a widely applied graph analysis method promising for graph classification, link prediction, node classification, and node clustering in psychoradiological studies. However, the interpretability for GNN models is even more challenging than other so-called black-box models because nodes and edges are often densely interconnected in a graph.

## Summary

Although AI methods have been used in diverse neuropsychiatric disorders in the development of psychoradiology, most applications are still at the level of simple case-control dichotomous classification. In mental illness clinical practice, physicians often face more complicated situations, such as comorbidity, differential diagnosis, and selection of a treatment option. There are also fundamental questions about the neurobiological validity of currently defined psychiatric syndromes and diagnostic nosology. Thus, rather than dividing psychiatric disorders into classical discrete categories such as current diagnostic categories, integrating multiple neurobiological dimensions of illness for defining discrete categories based on neurobiological features rather than traditional psychiatric diagnoses may be a better long-term approach for parsing enigmatic psychiatric disorders. Despite there being many challenges, psychoradiology had great promise for addressing clinical challenges in clinical psychiatry, with the help of establishing databases from multiple centers using a standard acquisition protocol, in conjunction with rapidly sophisticated algorithms and computer hardware. Therefore, there is a hope that AI tools can aid psychiatric physicians in diagnosis, prediction of prognosis, and treatment decision-making in the near to intermediate term. The combination of psychoradiology and AI has the potential to be gradually developed as a clinical examination method in psychiatric disorders, improving the efficiency, accuracy, and utility of diagnostic evaluations and treatment planning for psychiatric patients.
